# Exploring the Impact of Inhaled Corticosteroids on Endothelial Function in Chronic Obstructive Pulmonary Disease Patients Undergoing Pulmonary Rehabilitation

**DOI:** 10.3390/jcm13195749

**Published:** 2024-09-27

**Authors:** Pasquale Ambrosino, Claudio Candia, Claudia Merola, Carmen Lombardi, Costantino Mancusi, Maria Gabriella Matera, Mario Cazzola, Mauro Maniscalco

**Affiliations:** 1 Istituti Clinici Scientifici Maugeri IRCCS, Scientific Directorate of Telese Terme Institute, 82037 Telese Terme, Italy; pasquale.ambrosino@icsmaugeri.it; 2 Department of Clinical Medicine and Surgery, University of Naples “Federico II”, 80131 Naples, Italy; claudio.candia@unina.it; 3 Istituti Clinici Scientifici Maugeri IRCCS, Pulmonary Rehabilitation Unit of Telese Terme Institute, 82037 Telese Terme, Italy; claudia.merola@icsmaugeri.it (C.M.); carmen.lombardi@icsmaugeri.it (C.L.); 4 Department of Advanced Biomedical Sciences, University of Naples “Federico II”, 80131 Naples, Italy; costantino.mancusi@unina.it; 5 Department of Experimental Medicine, University of Campania “Luigi Vanvitelli”, 80138 Naples, Italy; mariagabriella.matera@unicampania.it; 6 Department of Experimental Medicine, University of Rome “Tor Vergata”, 00133 Rome, Italy; mario.cazzola@uniroma2.it

**Keywords:** pulmonary rehabilitation, exercise, disability, chronic obstructive pulmonary disease, endothelial function, outcome, occupational medicine

## Abstract

**Background**: Chronic obstructive pulmonary disease (COPD) is associated with subclinical atherosclerosis and endothelial dysfunction, which can be assessed non-invasively through flow-mediated dilation (FMD). In this study, we evaluated the potential impact of inhaled corticosteroid (ICS) therapy on FMD of COPD patients undergoing pulmonary rehabilitation (PR). **Methods**: Medical records of COPD patients undergoing FMD assessment upon admission to our Pulmonary Rehabilitation Unit were reviewed in this retrospective post hoc analysis. **Results**: A total of 46 patients with COPD (median age 71.5 years, 28.3% postmenopausal females) were included in the final analysis. Among these, 27 participants were currently receiving ICS therapy, while 19 were not. At baseline, the two groups showed no difference in the main clinical and functional variables. Similarly, no significant difference was observed in vascular reactivity parameters, with a median FMD of 3.12% (IQR: 2.23–4.45) in ICS users and 3.39% (IQR: 2.45–4.08) in ICS nonusers (*p* = 0.544). After PR, a significant improvement in the main rehabilitation and patient-reported outcomes was observed in all groups, with a significant improvement in FMD documented in both patients treated with steroids (from 3.12%; IQR: 2.23–4.45 to 4.77%; IQR: 3.25–5.63, *p* = 0.022) and in those who were not (from 3.39%; IQR: 2.45–4.08 to 5.04%; IQR: 3.98–6.06, *p* = 0.005). FMD changes were of comparable magnitude among groups. **Conclusions**: Our preliminary findings do not indicate a significant impact of medications containing ICS on the endothelial function of COPD patients, suggesting that the potential beneficial effect of PR on this surrogate marker of cardiovascular risk is independent of inhaled therapy.

## 1. Introduction

Chronic obstructive pulmonary disease (COPD) is a respiratory disorder characterized by fixed airflow limitation, exercise intolerance, acute exacerbations, and a gradual progression leading to respiratory failure [1]. This condition, often caused by chronic exposure to lung irritants such as smoking and pollution, significantly impairs the quality of life and imposes a substantial burden on healthcare systems [2]. An increasing body of evidence has underscored that COPD patients are also at a higher risk of developing cardiovascular (CV) diseases, including myocardial infarction, stroke, and heart failure [3]. It has been suggested that this risk in COPD may not be solely attributable to the shared exposure to traditional CV risk factors, like smoking, obesity, and sedentary lifestyle [4]. Instead, immune-mediated inflammation and oxidative stress are thought to play a crucial role in the development and progression of atherosclerosis in this clinical setting, due to their harmful effects on the vascular endothelium [5]. Thus, the disruption of endothelial barrier integrity in both systemic and pulmonary circulation may be a common pathogenic mechanism of many COPD manifestations, including the increased CV risk [5,6].

Endothelial dysfunction is, in fact, the earliest stage of atherosclerosis [7], with several methods being proposed to assess this parameter, both invasively and non-invasively [8]. In this regard, flow-mediated dilation (FMD) has been considered as a reliable technique and a surrogate marker of CV risk [9]. FMD involves the use of ultrasound to measure the dilation of the brachial artery following a period of occlusion and subsequent release, reflecting the endothelium’s ability to respond to increased blood flow [10]. Using this methodology, prior investigations have hinted at the possibility of a beneficial effect of multidisciplinary rehabilitation on the endothelial function of individuals with COPD, thereby potentially improving their CV risk profile [11]. Multidisciplinary rehabilitation programs and other exercise-based interventions often include nutritional counseling, psychological support, and educational programs [12,13,14], all of which can somehow impact overall CV health. In particular, it has been proposed that physical exercise, a cornerstone component of these programs, may enhance endothelial function by positively impacting systemic inflammation and oxidative stress [15]. In this scenario, pharmacological therapies, such as triple therapy combining inhaled corticosteroids (ICSs) with long-acting bronchodilators, may further reduce the systemic inflammation that contributes to endothelial dysfunction and atherosclerosis in COPD patients, thus potentially reducing overall and CV mortality [16] while reducing the frequency of exacerbations [17]. Accordingly, meta-analytical findings indicate a substantial risk reduction in CV disease, including heart attacks and strokes, associated with the use of ICS-containing drugs in COPD patients [18].

Therefore, this study aims to investigate the potential impact of concomitant ICS therapy on the FMD of COPD patients undergoing in-hospital pulmonary rehabilitation (PR).

## 2. Methods

We designed a retrospective post hoc analysis of prospectively collected data. A detailed protocol for data collection has been reported elsewhere and part of the baseline data have been previously published [11]. In brief, medical records of COPD patients undergoing FMD assessment upon admission to the Pulmonary Rehabilitation Unit of Istituti Clinici Scientifici Maugeri IRCCS, Telese Terme, Italy, between August 2020 and September 2022 were reviewed in this observational study. The inclusion criteria were as follows: age ≥ 40 years; confirmed diagnosis of COPD dating back at least one year; no acute exacerbation in the 4 weeks before admission and stable maintenance inhalatory therapy for at least the prior 3 months; and complete baseline FMD data gathered at study entry. Exclusions were applied to patients with major CV comorbidities, ongoing malignancies, any respiratory disease other than COPD, history of asthma in adulthood, recent (<12 months) severe acute respiratory syndrome coronavirus 2 (SARS-CoV-2) infection, or any history of severe-to-critical coronavirus disease 2019 (COVID-19) [9]. Where applicable, this study adhered to the Strengthening the Reporting of Observational Studies in Epidemiology (STROBE) guidelines [19] and complied with the Declaration of Helsinki by the World Medical Association. The Institutional Review Board of IRCCS Fondazione G. Pascale, Naples, Italy, reviewed and approved the protocol (No. 5.17OSS), and all participants provided written informed consent.

### 2.1. Study Protocol

Within 24 h from admission, relevant data on medical history and pharmacological therapy were collected for all study participants. In line with the principles outlined in the official American Thoracic Society/European Respiratory Society (ATS/ERS) statement [20], all included patients participated in a personalized, exercise-based multidisciplinary PR program, undergoing clinical, laboratory, and functional assessments at admission (T_0_) and, again, after completing the rehabilitation program (T_1_). 

### 2.2. Study Procedures

Whenever possible, study procedures were conducted at each time point, with participants in rooms maintained at a constant temperature of 23 °C. Under these conditions, fasting venous blood samples were drawn to assess various blood parameters, including creatinine (mg/dL), total cholesterol (mg/dL), triglycerides (mg/dL), glucose (mg/dL), and C-reactive protein (mg/dL). Following the recommendations of the European Society of Hypertension/European Society of Cardiology [21], systolic and diastolic blood pressure (SBP and DBP) were measured three times on separate occasions, with the average of these readings used as the final value.

Arterial oxygen tension (PaO_2_), arterial carbon dioxide tension (PaCO_2_), and power of hydrogen (pH) were measured in ambient air using a blood gas analyzer (ABL 825^®^ FLEX BGA, Radiometer Medical Aps, Copenhagen, Denmark) according to a standardized protocol [22]. Spirometry was performed on all COPD patients using an automated system (Vmax^®^ Encore, Vyasis Healthcare, Milan, Italy), in line with ATS/ERS guidelines [23,24]. Spirometry parameters, including forced expiratory volume in 1 s (FEV_1_) and forced vital capacity (FVC) values, were recorded as both absolute (liters) and percentages of predicted values (FEV_1_% and FVC%). The FEV_1_/FVC ratio was calculated as an indicator of airflow limitation.

To assess the impact of COPD on participants’ health, the COPD Assessment Test (CAT) [25] was utilized. Dyspnea severity was evaluated using the modified Medical Research Council (mMRC) dyspnea scale [26], while the Barthel index was used to measure daily living activities performance [27]. Additionally, the European Quality of Life (EuroQoL) scale was administered at each time point as a self-reported measure of health-related quality of life [28]. Functional exercise capacity was evaluated by measuring the six-minute walking distance (6MWD) in meters, following a predefined protocol [29].

### 2.3. Brachial Artery FMD Measurement

Brachial artery FMD was evaluated using ultrasound imaging, following the guidelines of the International Brachial Artery Reactivity Task Force [10]. A detailed protocol for this noninvasive vascular assessment has been described elsewhere [11]. In brief, measurements were taken after a 12 h fast and abstaining from smoking. Each examination was conducted after at least 10 min of rest in a supine position, with the use of a small head pillow being permitted and a blood pressure cuff placed on the forearm. A B-mode ultrasound machine (Vivid E95^®^, GE Healthcare, Chicago, IL, USA) and a linear vascular transducer with a 10 MHz frequency were used to image a longitudinal section of the brachial artery above the antecubital fossa. The brachial artery diameter (BAD) was measured at rest and following reactive hyperemia induced by forearm ischemia. To obtain a baseline image, the brachial artery was visualized for 1 min using pulsed Doppler before cuff inflation. The cuff was then inflated to 70 mmHg above the systolic pressure for 5 min. Following cuff deflation, BAD was registered for 4 min. The maximal post-occlusive diameter was used to calculate FMD as the percentage change in BAD during post-occlusive reactive hyperemia. The shear stress stimulus for FMD was determined by the shear rate area under the curve from cuff deflation to peak diameter (SR_AUC_), with shear rate calculated as (4 × blood velocity)/BAD. The total shear rate area under the curve (SR_AUC-TOT_) represented the complete post-occlusive reactive hyperemia over the 4 min following cuff deflation. Semi-automatic software (Cardiovascular Suite^®^ Version 3, FMD studio, QUIPU Srl, Pisa, Italy) was used to analyze the ultrasound images in real-time, thus calculating all vascular reactivity parameters. All recorded scans were independently analyzed by another ultrasound operator under blinded conditions to ensure strict quality control.

### 2.4. Pulmonary Rehabilitation

The interdisciplinary PR program for patients with COPD adhered to the latest ATS/ERS guidelines [20]. During the baseline assessment, a thorough evaluation of the extra-pulmonary features and comorbidities of COPD patients was conducted. This evaluation guided the application of various treatments, including physical exercise training, occupational therapy, dietary counseling, psychosocial counseling, and education. Physical exercise training was the cornerstone of the program. It involved exercises designed to strengthen muscle groups in the upper and lower extremities, flexibility exercises, treadmill walking, and stationary cycling. Supervised incremental aerobic exercise was planned, aimed at achieving at least 30 min of continuous activity at 50–75% of the maximal load [30]. Throughout the exercise sessions, pulse oximetry, blood pressure, and heart rate were continuously monitored. The daily activities lasted between 2 and 3 h, spread across 6 days per week, over a total period of 5 weeks. 

### 2.5. Statistical Analysis

Statistical analysis was performed using SPSS 29.0 (IBM, Chicago, IL, USA) with statistical significance set at *p* < 0.05. In brief, the Shapiro–Wilk test was employed to assess the normality of the distribution of continuous variables. In case of a Gaussian distribution, continuous variables were expressed as means ± standard deviation (SD), while in case of a skewed non-Gaussian distribution, they were reported as medians (interquartile range, IQR). Within-group comparisons between the two pre-specified time points were carried out with a paired Student’s *t*-test in case of normally distributed variables, and with a Wilcoxon Rank Test in case of skewed distributions. Comparisons between independent groups were performed using the unpaired Student’s *t*-test for normally distributed variables, and the Mann–Whitney U test for non-normally distributed variables. Categorical variables were reported as relative frequencies and compared using Pearson’s chi-squared test or Fisher’s exact test, as appropriate. 

## 3. Results

**Figure 1:**
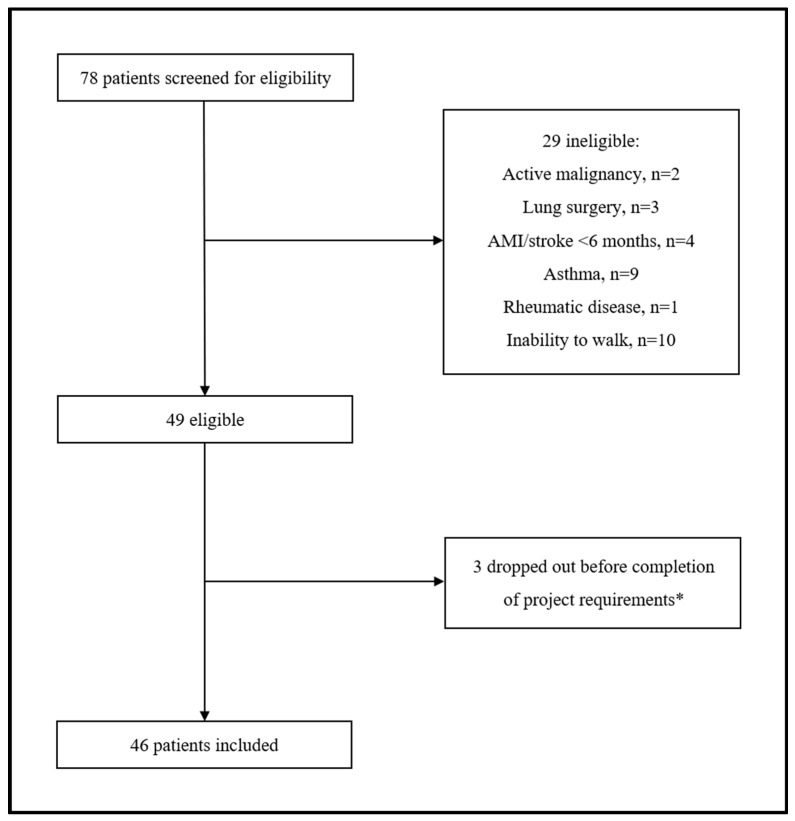
Flow chat of study participants. * Three patients transferred to another hospital as unable to attend/complete the rehabilitation program.

A total of 78 participants with COPD were screened for eligibility. Of them, 29 (37.2%) were excluded for various reasons related to the inclusion/exclusion criteria of our protocol. Among the 49 eligible patients, 3 (6.1%) withdrew before completion of the minimum project requirements. Therefore, 46 patients with COPD (median age 71.5 years, 28.3% postmenopausal females) were included in the final analysis (Figure 1).

All 46 participants were frequent exacerbators and, according to Global Initiative for Chronic Obstructive Lung Disease (GOLD) guidelines [31], were classified in Group E. Out of these, 27 were currently receiving ICS therapy, while 19 were not. The main clinical and functional characteristics of the two groups and changes from baseline (T_0_) to completion of the PR program (T_1_) are reported in Table 1.

At baseline, the two groups showed no difference in the main clinical and functional variables, except for a lower proportion of males among ICS users (*p* = 0.025) and higher peripheral saturation of oxygen (*p* = 0.022), as well as arterial oxygen partial pressure (*p* = 0.012). Similarly, no significant difference was observed between the two groups with regards to vascular reactivity parameters, with a median FMD of 3.12% (IQR: 2.23–4.45) in ICS users and 3.39% (IQR: 2.45–4.08) in ICS nonusers (*p* = 0.544) at T_0_.

**Figure 2:**
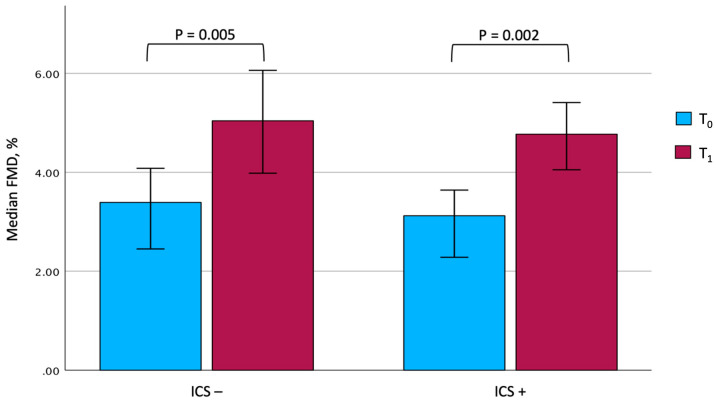
Changes in FMD values in the two groups. The comparison between the two groups at T_0_ and T_1_ was not significant. Data are expressed as median and 95% confidence interval. Abbreviations: FMD, flow-mediated dilation. T_0_, baseline. T_1_, follow-up. ICS, inhaled corticosteroids.

At the end of the rehabilitation program, due to technical issues, complete information on vascular reactivity could be gathered for only 25 out of 27 patients under ICS and 15 out of 19 patients not taking them. Although some differences were observed between the two groups, it is interesting to note that both showed a significant improvement in the main rehabilitation and patient-reported outcomes. In particular, the 6MWD significantly improved after PR (from 166.0 ± 73.1 m to 242.1 ± 88.8 m, *p* < 0.001, among ICS users, and from 166.5 ± 74.4 m to 246.4 ± 79.1 m, *p* < 0.001, among ICS nonusers), with comparable mean changes between the two groups (81.8 ± 42.9 m in ICS users vs. 79.9 ± 54.0 m in ICS nonusers, *p* = 0.900). Similarly, a significant improvement in FMD could be documented in both patients treated with steroids (from 3.12%; IQR: 2.23–4.45 to 4.77%; IQR: 3.25–5.63, *p* = 0.022) and in those who were not (from 3.39%; IQR: 2.45–4.08 to 5.04%; IQR: 3.98–6.06, *p* = 0.005), with changes of comparable magnitude (Figure 2). In contrast, no significant changes in BAD, SR_AUC_, and SR_AUC-TOT_ were documented in both groups. After completing PR, it is remarkable that ICS users showed a significantly higher SR_AUC_ compared with nonusers (*p* = 0.016).

## 4. Discussion

Albeit preliminary, our findings do not suggest a significant impact of ICS-containing medications on the endothelial function of COPD patients, while indicating that the potential beneficial effect of rehabilitation on this CV risk marker may occur independently of the prescribed therapy.

This report thus contributes to a scientific landscape that consistently shows that COPD is burdened by an elevated prevalence of CV comorbidities, which account for 30% of its excess mortality [32]. The latest GOLD document stressed the crucial importance of preventing acute exacerbations [31], which have been linked to an increase in CV risk in such patients [33]. In this regard, a mounting body of evidence has been showing that triple inhaled therapy, comprising ICS combined with long-acting bronchodilators, is more efficient than dual therapy with long-acting bronchodilators alone in reducing the frequency of exacerbations [17], thus improving symptoms and reducing both overall and CV mortality [16]. Such benefits deriving from the use of triple therapy in COPD patients could be explained by the presence of ICSs, which reduce bronchial inflammation and decrease the amount of circulant reactive oxide species (ROS), which have been linked to endothelial dysfunction [34].

Endothelial dysfunction is considered the earliest pathogenetic mechanism of CV disease [7], representing a marker of subclinical atherosclerosis [9] and even an attractive therapeutic target [35]. In our previous study [11], we demonstrated that endothelial function assessed by FMD could significantly and consistently improve in COPD patients after an intensive PR program, independently of most traditional CV risk factors, except for hypercholesterolemia. Given the evidence of CV risk reduction associated with steroid therapy in COPD [18], we hypothesized that ICS may somehow impact the FMD of COPD patients undergoing PR. The fact that our results indicate otherwise suggests that the positive effects of PR on endothelial function and, potentially, on CV risk, may also be independent of steroid therapy. Numerous studies have previously documented the positive impact of rehabilitation and exercise-based approaches on endothelial function, arterial stiffness, and various surrogate markers of CV risk, not only in respiratory diseases, but also in other clinical settings [36,37,38,39]. Therefore, it could be hypothesized that the effect of steroids might have been somewhat “diluted” by an exercise-based intervention, which has already demonstrated its strength in improving endothelial function. In other words, ICS may not simply provide additional support in terms of CV risk reduction in adjunct to PR. Another aspect to consider when interpreting the results of our post hoc analysis is the specific population enrolled, characterized by a group of COPD patients with severe disease and a high number of exacerbations per year. This might lead us to believe that our results are not generalizable to all stages of the disease and that the inflammatory state of our hyper-selected population may account for the above results. It is important to highlight that, beyond the effects of steroids on CV risk during rehabilitation, our findings also indicate that steroids do not impact baseline FMD values. Given the small sample size, we believe this does not contradict the epidemiological evidence showing a reduction in CV risk associated with steroids in COPD [18], but rather aligns with meta-analytic data suggesting that, at the very least, steroids do not worsen this risk [40].

Some important limitations of this retrospective post hoc analysis should be outlined, in addition to those of the original protocol that have been extensively described elsewhere [11]. Firstly, our analyses were conducted after data collection and may not align with the original study design, which can introduce biases. Thus, uncontrolled confounding factors may influence our results and, as the analysis was not pre-specified, no formal sample size calculation was performed. Moreover, there was a high risk of Type I errors due to multiple comparisons, which could have led to false positives or negatives. Finally, the findings of our analyses may not be generalizable beyond the study sample due to its specific context and limitations.

## 5. Conclusions

Overall, our results suggest that ICSs do not have a significant impact on endothelial function in patients with COPD and, most importantly, they indicate that the potential positive effect of PR on this CV risk marker is not influenced by concurrent steroid therapy. While acknowledging the preliminary nature of these findings and the considerable limitations of our analysis, our results do not rule out the potential CV benefits of corticosteroids, but rather suggest that, at the very least, triple inhaler therapy does not have a negative effect on CV risk. Furthermore, our findings reinforce the potential effectiveness of exercise-based interventions in improving the CV risk profile of COPD patients, while indicating that the potential beneficial effect of rehabilitation on endothelial function may occur independently of the prescribed therapy.

## Figures and Tables

**Table 1:** Main clinical and functional characteristics of chronic obstructive pulmonary disease (COPD) patients undergoing pulmonary rehabilitation, stratified and compared by inhaled corticosteroid (ICS) therapy.

Variable	ICS Users			ICS Nonusers			Users vs. Nonusers (*p*-Value)	
	**T_0_**	**T_1_**	***p*-Value**	**T_0_**	**T_1_**	***p*-Value**	**T_0_**	**T_1_**
	27	25	-	19	15	-	-	-
**Demographics**								
Males, *n* (%)	16 (59.3)	15 (60.0)	-	17 (89.5)	13 (86.7)	-	**0.025**	-
Age, years	71.6 ± 7.3	-	-	71.2 ± 8.9	-	-	0.845	-
BMI, m^2^/kg	28.1 ± 5.5	26.9 ± 5.0	**0.009**	27.6 ± 7.0	27.1 ± 6.9	0.463	0.401	0.456
**Clinical history**								
Active smokers, *n* (%)	5 (18.5)	-	-	1 (5.2)	-	-	0.195	-
Exacerbations/year, *n*	2.0 ± 1.6	-	-	1.7 ± 1.1	-	-	0.459	-
**Clinical parameters**								
SBP, mmHg	120.0 (120.0–130.0)	120.0 (117.5–130.0)	**0.042**	130.0 (120.0–140.0)	120.0 (120.0–130.0)	**0.011**	0.129	0.339
DBP, mmHg	80.0 (70.0–80.0)	75.0 (70.0–80.0)	0.516	80.0 (80.0–80.0)	80.0 (70.0–80.0)	**0.002**	**0.011**	0.741
SpO_2_, %	94.0 (92.0–96.0)	94.0 (90.5–95.0)	0.350	93.0 (86.3–94.8)	94.0 (89.5–95.0)	0.050	**0.022**	0.834
**Laboratory**								
Total cholesterol, mg/dL	165.9 ± 41.9	133.1 ± 34.3	0.062	161.5 ± 34.7	128.1 ± 38.1	0.138	0.366	0.391
Triglycerides, mg/dL	106.0 (79.0–139.0)	126.0 (94.5–135.8)	0.333	118.0 (77.0–153.0)	115.0 (70.0–183.0)	0.735	0.979	0.417
Serum glucose, mg/dL	94.0 (78.0–109.0)	88.5 (80.5–91.75)	**0.005**	91.0 (78.0–99.0)	86.0 (74.0–96.0)	0.612	0.590	1.000
Creatinine, mg/dL	0.82 (0.62–1.06)	1.14 (0.78–1.24)	0.075	1.00 (0.71–1.13)	0.96 (0.75–1.76)	0.262	0.248	0.657
C-reactive protein, mg/L	2.9 (1.9–6.2)	10.6 (2.4–69.2)	0.080	7.3 (3.3–20.7)	4.2 (2.5–30.3)	0.500	0.069	0.366
**Patient-reported outcomes**								
CAT score	27.0 (24.5–29.0)	13.5 (10.5–17.5)	**0.002**	28.0 (24.0–29.8)	17.0 (10.0–17.5)	0.068	0.703	0.269
ΔCAT	-	−13.1 ± 4.5	-	-	−13.5 ± 3.1	-	-	0.867
mMRC score	4.0 (0–4.0)	2.0 (2.0–2.0)	**<0.001**	4.0 (0–4.0)	2.0 (2.0–2.0)	**<0.001**	0.441	0.367
Δ mMRC	-	−2.0 ± 0.7	-	-	−2.0 ± 0.4	-	-	0.952
**Arteral blood gases**								
pH	7.39 (7.37–7.43)	7.41 (7.39–7.43)	0.418	7.42 (7.38–7.45)	7.43 (7.39–7.44)	0.858	0.074	0.289
PaO_2_, mmHg	72.8 (60.6–79.7)	66.0 (56.0–76.2)	0.159	57.0 (52.0–66.0)	66.0 (51.4–69.0)	0.091	**0.012**	0.389
PaCO_2_, mmHg	42.5 (39.3–47.4)	44.0 (38.1–49.0)	0.297	41.0 (38.2–45.2)	40.0 (36.7–51.1)	0.372	0.633	0.506
**Lung function**								
FEV_1_, L	1.14 ± 0.43	1.19 ± 0.43	0.124	1.01 ± 0.30	1.23 ± 0.47	**0.003**	0.142	0.400
ΔFEV_1_, L	-	0.01 (−0.03–0.23)	-	-	0.21 (0.05–0.29)	-	-	0.076
FEV_1_%, % predicted	43.0 (31.5–57.0)	46.5 (34.8–58.5)	**0.035**	39.0 (29.3–48.5)	48.0 (32.0–66.0)	**0.003**	0.330	0.640
ΔFEV_1_%, % predicted	-	3.0 (−0.5–11.0)	-	-	8.0 (3.5–13.0)	-	-	0.185
FVC, L	1.92 (1.72–2.70)	2.33 (1.78–2.71)	0.068	2.03 (1.64–2.34)	2.23 (1.83–2.94)	**0.004**	0.676	0.736
ΔFVC, L	-	0.14 (−0.06–0.52)	-	-	0.25 (0.11–0.91)	-	-	0.148
FVC%, % predicted	66.7 ± 18.3	71.9 ± 23.1	**0.032**	59.9 ± 16.8	72.3 ± 26.4	**0.012**	0.226	0.955
ΔFVC%, % predicted	-	6.0 (−1.0–19.0)	-	-	7.5 (2.5–13.3)	-	-	0.592
FEV_1_/FVC	47.0 (41.0–60.0)	52.0 (42.8–57.3)	0.571	45.5 (40.0–57.5)	51.0 (42.0–68.0)	0.867	0.631	0.904
**Rehabilitation outcomes**								
6MWD, m	166.0 ± 73.1	242.1 ± 88.8	**<0.001**	166.5 ± 74.4	246.4 ± 79.1	**<0.001**	0.984	0.869
Δ6MWD, m	-	81.8 ± 42.9	-	-	79.9 ± 54.0	-	-	0.900
Barthel Index	75.0 (65.0–80.0)	94.0 (83.3–95.0)	0.061	70.0 (31.8–79.8)	85.0 (48.0–92.5)	**0.017**	0.269	0.212
ΔBarthel Index	-	16.0 (0–21.0)	-	-	18.0 (2.5–27.0)	-	-	0.668
EuroQOL score	50.0 (42.5–50.0)	80.0 (75.0–80.0)	**<0.001**	42.5 (40.0–50.0)	75.0 (70.0–80.0)	**0.003**	0.142	0.740
ΔEuroQOL	-	30.2 ± 6.8	-	-	30.1 ± 6.3	-	-	0.787
**Vascular reactivity**								
FMD, %	3.12 (2.23–4.45)	4.77 (3.25–5.63)	**0.002**	3.39 (2.45–4.08)	5.04 (3.98–6.06)	**0.005**	0.544	0.318
ΔFMD, %	-	1.18 (−0.14–2.56)	-	-	1.99 (−0.14–3.13)	-	-	0.422
BAD, mm	4.00 ± 0.86	3.98 ± 0.74	0.914	4.18 ± 0.60	4.12 ± 0.48	0.072	0.366	0.609
SR_AUC_	27,248 (14,143–94,044)	32,280 (15,314–73,747)	0.701	18,862 (9324–33,558)	17,211.4 (11,831–29,307)	0.334	0.084	**0.016**
SR_AUC-TOT_	77,457 (37,493–105,634)	48,134 (32,501–97,424)	0.439	54,588 (41,823–70,070)	30,897 (22,962–50,798)	0.256	0.126	0.072

Abbreviations: T_0_, baseline. T_1_, follow-up. *n*, number. BMI, body mass index. SBP, systolic blood pressure. DBP, diastolic blood pressure. SpO_2_, peripheral oxygen saturation. CAT, COPD assessment test. mMRC, modified Medical Research Council scale for dyspnea. Δ, change from baseline. PaO_2_, arterial partial pressure of oxygen. PaCO_2_, arterial partial pressure of carbon dioxide. FEV_1_, forced expiratory volume in the first second. FVC, forced vital capacity. 6MWD, 6 min walking distance. EuroQOL, European Quality of Life questionnaire. FMD, flow-mediated dilation. BAD, brachial artery diameter. SR_AUC_, shear rate area under the curve to peak diameter. SR_AUC-TOT_, total shear rate area under the curve.

## Data Availability

The data supporting the findings of this study are available from the corresponding authors upon reasonable request due to privacy/ethical restrictions.
